# The pericoronary adipose tissue attenuation in CT strongly depends on kernels and iterative reconstructions

**DOI:** 10.1007/s00330-024-11132-5

**Published:** 2024-10-18

**Authors:** Costanza Lisi, Konstantin Klambauer, Lukas J. Moser, Victor Mergen, Robert Manka, Thomas Flohr, Matthias Eberhard, Hatem Alkadhi

**Affiliations:** 1 Diagnostic and Interventional Radiology, University Hospital Zurich, University of Zurich, Zurich, Switzerland; 2 Department of Biomedical Sciences, Humanitas University, via Rita Levi Montalcini 4, 20090 Pieve Emanuele Milan, Italy; 3 IRCCS Humanitas Research Hospital, Via Manzoni 56, 20089 Rozzano, Milan Italy

**Keywords:** Computed tomography angiography, Pericoronary adipose tissue, Inflammation, Image reconstruction

## Abstract

**Objectives:**

To investigate the influence of kernels and iterative reconstructions on pericoronary adipose tissue (PCAT) attenuation in coronary CT angiography (CCTA).

**Materials and methods:**

Twenty otherwise healthy subjects (16 females; median age 52 years) with atypical chest pain, low risk of coronary artery disease (CAD), and without CAD in photon-counting detector CCTA were included. Images were reconstructed with a quantitative smooth (Qr36) and three vascular kernels of increasing sharpness levels (Bv36, Bv44, Bv56). Quantum iterative reconstruction (QIR) was either switched-off (QIRoff) or was used with strength levels 2 and 4. The fat-attenuation-index (FAI) of the PCAT surrounding the right coronary artery was calculated in each dataset. Histograms of FAI measurements were created. Intra- and inter-reader agreements were determined. A CT edge phantom was used to determine the edge spread function (ESF) for the same datasets.

**Results:**

Intra- and inter-reader agreement of FAI was excellent (intra-class correlation coefficient = 0.99 and 0.98, respectively). Significant differences in FAI were observed depending on the kernel and iterative reconstruction strength level (each, *p* < 0.001), with considerable inter-individual variation up to 34 HU and intra-individual variation up to 33 HU, depending on kernels and iterative reconstruction levels. The ESFs showed a reduced range of edge-smoothing with increasing kernel sharpness, causing an FAI decrease. Histogram analyses revealed a narrower peak of PCAT values with increasing iterative reconstruction levels, causing a FAI increase.

**Conclusions:**

PCAT attenuation determined with CCTA heavily depends on kernels and iterative reconstruction levels both within and across subjects. Standardization of CT reconstruction parameters is mandatory for FAI studies to enable meaningful interpretations.

**Key Points:**

***Question*** *Do kernels and iterative reconstructions influence pericoronary adipose tissue (PCAT) attenuation in coronary CT angiography (CCTA)?*

***Findings*** *Significant differences in fat-attenuation-index (FAI) were observed depending on the kernel and iterative reconstruction strength level with considerable inter- and intra-individual variation.*

***Clinical relevance*** *PCAT attenuation heavily depends on kernels and iterative reconstructions requiring CT reconstruction parameter standardization to enable meaningful interpretations of fat-attenuation differences across subjects.*

## Introduction

Pericoronary adipose tissue (PCAT) is hormonally active and exerts various effects on the coronary artery wall by releasing endocrine, paracrine, and vasocrine substances [1]. Vice versa, the coronary artery endothelium affects PCAT by releasing neuro-hormonal mediators [2]. Thus, PCAT is considered important in the process of coronary inflammation eventually leading to coronary atherosclerosis.

Cardiac computed tomography (CT) has been suggested as an imaging tool to quantify coronary inflammation by demonstrating a shift of PCAT attenuation toward higher Hounsfield units (HUs) [3, 4]. PCAT is usually defined as the average attenuation in the range of −190 HU to −30 HU in a volume-of-interest (VOI) surrounding the proximal right coronary artery (RCA) [5]. Whereas some studies demonstrated PCAT attenuation determined with CT to enable cardiovascular risk stratification [6], diagnosis and prognosis of ischemic heart disease [7, 8], as well as cardiac mortality assessment [9], others could not confirm these results [10–12]. Ma et al [10] found no difference in PCAT attenuation between patients with and without coronary artery disease (CAD), and Pandey et al [11] demonstrated that patients with obstructive CAD had even lower PCAT attenuation than those without. Boussoussou et al [12] found no correlation between PCAT and CAD after correcting results for image acquisition parameters and patient characteristics. The latter study suggested that these divergent results in the literature may be explained by variable, non-standardized cardiac CT protocols [12].

As a matter of fact, CT scanner types, scan and contrast media protocols, and image reconstruction parameters differ considerably in the PCAT literature [13–23]. In previous studies, authors utilized different X-ray tube voltages [13–23], polychromatic *versus* virtual monoenergetic images [16, 17], different kernels, and variable types and degrees of iterative reconstructions [4, 8, 9, 18]. All these factors are known to affect attenuation and noise and hence, might affect PCAT attenuation values as well [13].

The aim of our study was to investigate the influence of kernels and iterative reconstructions on PCAT attenuation in a cohort of subjects without evidence of CAD who underwent coronary photon-counting detector CT angiography.

## Materials and methods

### Patient population

This retrospective study was performed at a tertiary academic hospital and had institutional review board and ethics committee agreement. All subjects provided general consent for further use of their data for anonymized research. Consecutive subjects referred for cardiac computed tomography angiography (CCTA) between April and May 2024 were screened for inclusion in the study based on the following criteria: otherwise healthy subjects undergoing CCTA, having a low risk of CAD, showing no evidence of coronary atherosclerosis in CCTA, and having an excellent image quality of CCTA. Healthy subjects were included to minimize potential differences in PCAT caused by underlying disease. CCTA was performed to rule out CAD in atypical chest pain in 12 subjects (60%), atypical chest pain and non-specific electrocardiography (ECG) changes in 3 subjects (15%), and exertional dyspnoea in the remaining (25%).

Twenty subjects with low risk of CAD, without coronary calcifications (coronary artery calcium score of 0 in each), without non-calcified plaques, and without coronary stenosis in CCTA were included (16 females; 4 males; median age 52 years (IQR 48–61 years), median body mass index 25 kg/m^2^ (IQR 23–27 kg/m^2^) (Table 1). The subjects had no other comorbidities, including diabetes, oncologic, rheumatological, pulmonary, or hematological disease.

**Table 1:** Subject demographics

Characteristic	*n* = 20
Sex	
Female	16 (80%)
Male	4 (20%)
Age (years)	52 (48–61)
Body weight (kg)	70 (65–88)
Body mass index (kg/m^2^)	25 (23–27)
Average heart rate during acquisition (bpm)	69 (63–82)
Medical history	
Arterial hypertension	3 (15%)
Diabetes	0 (0%)
Dyslipidemia	10 (50%)
Smoking history	3 (15%)
Presenting symptoms/clinical question	
Atypical chest pain	12 (60%)
Atypical chest pain & non-specific ECG changes	3 (15%)
Exertional dyspnea	5 (25%)
Other comorbidities	0 (0%)
Average coronary artery calcium score	0

Note: Unless otherwise indicated, data are median and IQR or number of subjects with percentages in parentheses

*bmp* betas per minute, *ECG* electrocardiography, *IQR* interquartile range, *n* number of subjects

### CT data acquisition and image reconstruction

CCTA was acquired in the prospectively ECG-triggered spectral mode (quantum Plus mode) on a dual-source photon-counting detector CT scanner (NAEOTOM Alpha, software version VB10; Siemens Healthineers AG). Detector collimation was 144 × 0.4 mm; tube voltage was 120 kVp, and automated tube current-modulation (CARE Dose4D, Siemens) was applied with an image quality level of 64. Gantry rotation time was 0.25 s, with a temporal resolution of 66 milliseconds. The median volume CT dose index was 31.2 mGy (interquartile range (IQR), 23.9–40.1 mGy). A triphasic contrast media protocol was administered (45–80 mL iopromide, Ultravist 370 mg I/mL; Bayer Healthcare); flow rates ranged from 3.2 mL/s to 6.0 mL/s, depending on subject’s body mass index. Bolus tracking guided the acquisition, with a threshold of 140 HU in the ascending aorta at 90 kVp. Sublingual nitroglycerin (2.5 mg isosorbide dinitrate) was administered to all subjects prior to the examination. No beta-blocker medication was given. Reconstruction field-of-views were 200 × 200 mm using a matrix of 512 × 512 pixels. Slice thickness was 0.6 mm with a 0.3 mm increment. Monoenergetic reconstructions were performed at a level of 55 keV. An algorithm for reduction of possible stair-step artefacts was applied (*ZeeFree*, Siemens) [24]. The single best phase showing the least motion artefacts was selected and was reconstructed for each patient with the following kernels:

- A quantitative soft tissue kernel Qr36 (smooth) with moderate sharpness (50% value of the modulation transfer function (MTF) 3.38 lp/cm), which is recommended for quantitative evaluation of spectral data;
- Three vascular soft tissue kernels with increasing sharpness levels:
  - Bv36 (smooth) with a 50% MTF of 3.38 lp/cm, similar to Qr36,
  - Bv44 (medium) with a 50% MTF of 4.62 lp/cm, and
  - Bv56 (sharp) with a 50% MTF of 7.39 lp/cm.

Vascular kernels are characterized by an edge enhancement that increases with the strength level of the iterative reconstruction to better delineate the vessel edges. Kernels with the sharpness characteristics of Bv36 were routinely used for CCTA with older CT scanners. Bv44 is the standard kernel for CCTA in photon-counting detector CT [25, 26], and Bv56 is used for ultra-high-resolution CCTA [27].

For each of the above-listed kernels, image data were reconstructed without quantum iterative reconstruction (QIR off) and with QIR at strength level 2 and level 4 [28], leading to 12 datasets for each subject (a total of 240 datasets for further analysis). QIR is an iterative reconstruction algorithm that corrects for geometric cone beam artifacts and performs a statistical optimization of spectral data, where QIR off is a reconstruction in which minimally possible statistical optimization is obtained with respect to standard weighted filtered back projection, while QIR strength levels 2 and 4 include an additional optimization in terms of a progressively reduced noise [28].

### Pericoronary adipose tissue analysis

**Fig. 1:**
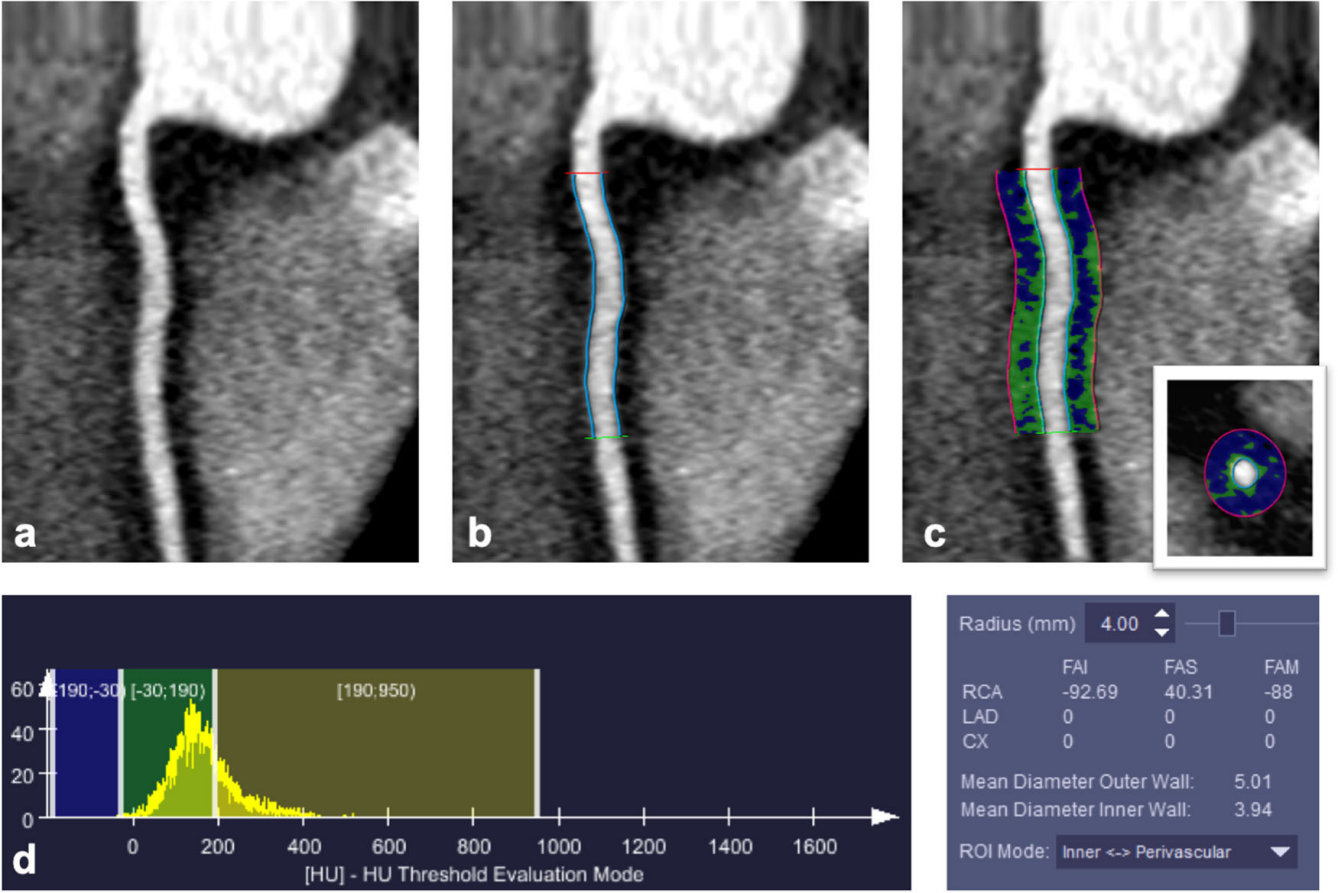
Illustration of the semiautomated pericoronary adipose tissue analysis software approach in a 48-year-old male subject undergoing CCTA for atypical chest pain showing no evidence of CAD. Curved reformation of the proximal right coronary artery (RCA) (**a**), segmentation of proximal 40 mm RCA segment length by proximal (red) and distal markers (green) with vessel wall automatic contouring (light blue lines) (**b**). Pericoronary adipose tissue definition (**c**) in curved and short-axis reformats (insert in **c**) is the tissue within a radial distance from the inner vessel wall equal to the vessel diameter. Pericoronary adipose tissue identification (**c**) in the attenuation range from −190 HU to −30 HU (blu voxels) with corresponding histogram (**d**, left side). Quantitative pericoronary fat tissue analysis results (**d**, right side) indicate a FAI value of 93 HU in this subject and in this individual reconstruction (Bv36, QIRoff)

PCAT analysis was performed for each dataset using semiautomatic software (CT Coronary Plaque Analysis, software version 5.0.3, Siemens Healthineers AG) (Fig. 1). The proximal 50 mm of the RCA was manually defined by one reader ((C.L.), resident with three years of experience in cardiovascular imaging). The ostial 10 mm of the RCA was excluded to avoid the effect of the aortic wall, as previously described [29]. The software then automatically detected the outer and inner vessel walls, and manual correction was performed if required. Pericoronary tissue was defined as the adipose tissue within a radial distance from the inner vessel wall equal to the vessel diameter, and PCAT was identified in the attenuation range from −190 to −30 HU [29].

Perivascular analysis output included the fat-attenuation index (FAI) value, representing the mean attenuation inside the VOI, and the standard deviation of the HU values, which represents noise. All FAI values were noted, and histograms were extracted from each PCAT VOI for each dataset. PCAT semiautomatic analysis of the proximal RCA took an average of 3–5 min per specific reconstruction. PCAT analysis was performed for each dataset twice by the same blinded member of the study team (C.L.) to determine the intra-reader agreement after three weeks to avoid recall bias. Inter-reader agreement was determined by performing the analyses by another blinded member of the study team ((K.K.), a resident with three years of experience in cardiovascular imaging). Both readers applied the pericoronary fat analysis protocol described above over the proximal 50 mm of RCA, with the ostial 10 mm sparing to avoid ascending aortic wall influence and to grant reproducibility of the measurements.

### Image noise and vessel attenuation assessment

Image noise was determined as the standard deviation of attenuation in the VOI surrounding the RCA in all subjects and in each reconstruction by one reader (C.L.). Vessel attenuation was defined as attenuation in a region-of-interest in the ascending aorta at the level of the ostium of the RCA, copy/pasted across all datasets.

### Edge spread function

A CT water phantom (QRM) with 18 cm diameter including an inserted Teflon block with inclined edges was scanned using the same CT scan protocol and reconstructions detailed in the paragraph above. Edge spread functions (ESF) for the different reconstructions were determined with an inhouse Matlab program (The Mathworkses). The ESF shows how an edge, in our case, the vessel edge, is displayed in the CT image and how it depends on the convolution kernel and the strength of the iterative reconstruction.

### Statistical analysis

Quantitative variables are expressed as mean ± standard deviation or median and IQR, as applicable. Qualitative variables are reported as counts or percentages. Intra-class correlation coefficients (ICCs) were used to calculate intra- and inter-reader agreement with 95% confidence intervals (CI). Intra- and inter-reader agreement was classified using the following scale: 0–0.20, poor agreement; 0.21–0.40, fair agreement; 0.41–0.60, moderate agreement; 0.61–0.80, substantial agreement; and 0.81–1.00, excellent agreement. Statistical analyses involved a two-way repeated measures analysis of variance (ANOVA) to assess the effects of kernel and iterative reconstruction strength levels on the FAI and noise. The ANOVA model included the main effects of the kernel and iterative reconstruction strength levels, as well as their interaction, with each patient serving as their own control. Post-hoc pair-wise comparisons were conducted using Tukey’s Honest Significant Difference (HSD) test. Two-tailed *p*-values below 0.05 were considered to infer significance. All analyses, including ANOVA and post-hoc tests, were conducted using R statistical software (R, version 4.4.0; R Foundation).

## Results

Twenty subjects with low risk of CAD, without coronary calcifications (coronary artery calcium score of 0 in each), without non-calcified plaques, and without coronary stenoses in CCTA were included. Patient demographics are presented in Table 1.

### Pericoronary adipose tissue analysis

Intra-reader agreement of FAI values was excellent, with an ICC of 0.99 (0.982–0.992 95% CI; *p* < 0.001). Inter-reader agreement was also excellent, with an ICC of 0.98 (0.976–0.988 95% CI; *p* < 0.001). Based on the excellent inter-reader agreement, further analyses were performed using the average measurements of one reader.

**Fig. 2:**
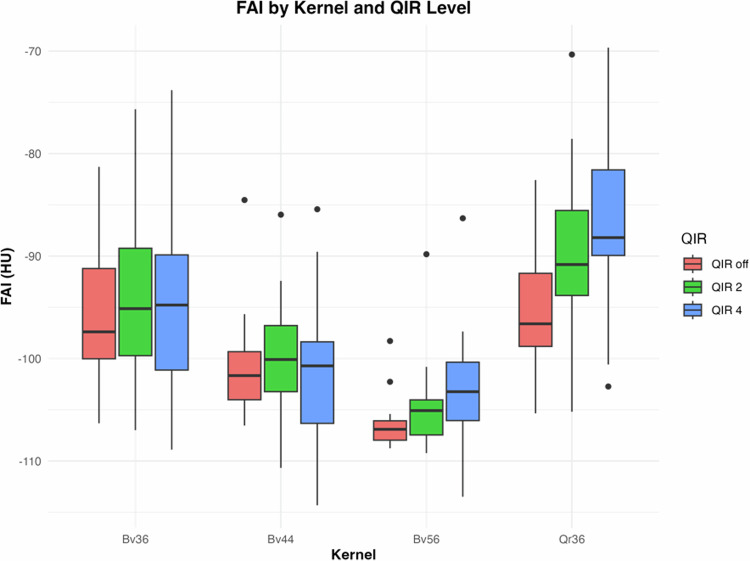
Boxplots demonstrating the FAI (median and standard deviation) by kernel and iterative reconstruction strength level. Note the considerable variability of FAI values both within and between subjects

FAI values differed across different subjects using the same reconstructions (Table 2). Inter-individually, the largest difference between the maximum and minimum FAI was found in reconstructions with a vascular smooth kernel (Bv36) and QIR 4 (delta 34 HU), while the smallest inter-individual differences were found in reconstructions with a sharp vascular kernel (Bv56) without iterative reconstruction (QIR off, delta 10 HU) (Fig. 2).

**Table 2:** Mean pericoronary FAI ± SD (ranges) for different kernels and iterative reconstruction strength levels

Subjects (*n* = 20)	Vessel radius (mm)	Qr36			Bv36			Bv44			Bv56		
		*QIR off* (HU)	*QIR 2* (HU)	*QIR 4* (HU)	*QIR off* (HU)	*QIR 2* (HU)	*QIR 4* (HU)	*QIR off* (HU)	*QIR 2* (HU)	*QIR 4* (HU)	*QIR off* (HU)	*QIR 2* (HU)	*QIR 4* (HU)
	3.7 ± 0.8	−95 ± 5 (−105; −83)	−90 ± 8 (−103; −70)	−87 ± 68 (−101; −70)	−96 ± 6 (−106; −81)	−95 ± 8 (−107; −76)	−95 ± 9 (−108; −74)	−101 ± 5 (−106; −85)	−100 ± 6 (−110; −86)	−101 ± 8 (−114; −85)	−106 ± 2 (−109; −98)	−105 ± 4 (−109; −90)	−103 ± 6 (−113; −86)

*FAI* fat-attenuation index, *HU* Hounsfield unit, *IQR* interquartile range, *QIR* quantum iterative reconstruction, *RCA* right coronary artery, *SD* standard deviation

FAI values differed among kernels and the presence and strength levels of iterative reconstruction (see Fig. 2 and Table 2). Overall, the lowest FAI values were found in reconstructions with a sharp vascular kernel (Bv56) without iterative reconstruction (QIR off) in most (75%) subjects (mean FAI − 106 ± 2 HU). The highest FAI values were found mostly (95% of subjects) in reconstructions with a quantitative smooth kernel (Qr36) and QIR at a strength level of 4 (mean FAI − 87 ± 9 HU) (see Fig. 2), with an average difference between maximum and minimum FAI values of 19 HU.

Intra-individually, the largest differences between the maximum and minimum FAI were found in patient no. 13 (delta 33 HU), and smallest intra-individual differences were found in patient no. 7 (delta 11 HU).

Effect of iterative reconstruction at the same kernel on FAI: largest differences in FAI values were demonstrated with the vascular smooth kernel (Bv36) (increase by 12 HU from QIR off to QIR4), while the smallest differences were found for the medium vascular kernel (Bv44) (increase by 0.6 HU from QIR off to QIR4). ANOVA indicated significant main effects of iterative reconstruction strength level on FAI (*p* < 0.001). Post-hoc tests revealed significant differences between QIR off and QIR2, as well as between QIR off and QIR4, particularly for the Qr36 kernel (Table 3 and Fig. 2).

**Table 3:** Comparison of FAI across kernels and iterative reconstruction strength levels

Kernel	QIR off (HU)	QIR2 (HU)	HSD^a^	QIR4 (HU)	HSD^b^	ANOVA^c^
Bv36	−96.42 ± 6.34	−94.74 ± 7.95	0.111	−94.75 ± 9.02	0.755	0.740
Bv44	−100.86 ± 4.99	−100.25 ± 5.94	0.994	−101.4 ± 7.61	> 0.999	0.845
Bv56	−106.43 ± 2.4	−104.58 ± 4.21	0.025	−103.36 ± 6.08	0.124	0.102
Qr36	−95.17 ± 5.58	−90.09 ± 8.36	< 0.001	−86.65 ± 8.68	< 0.001	0.004
ANOVA^c^	< 0.001	< 0.001	^-^	< 0.001	-	-
**QIR level**	**Mean FAI of all kernels (HU)**		**HSD**^**a**^			
QIR off	−97.23 ± 7.32		-			
QIR 2	−96.69 ± 9.83		0.058			
QIR 4	−90.64 ± 8.32		0.013			
**Kernel**	**Mean FAI of all QIR levels (HU)**		**HSD**^**b**^			
Bv36	−95.3 ± 7.76		-			
Bv44	−100.83 ± 6.1		< 0.001			
Bv56	−104.79 ± 4.59		< 0.001			
Qr36	−90.64 ± 8.32		< 0.001			

Note: values are mean and standard deviation unless otherwise specified

*ANOVA* analysis of variance, *FAI* fat-attenuation index, *HSD* honestly significant difference test, *HU* Hounsfield units, *QIR* quantum iterative reconstruction

^a^ QIR off vs. QIR2, QIR off vs. QIR4

^b^ Bv36 vs. Bv44, Bv36. vs. Bv56, Bv36 vs. Qr36

^a,b^ *p*-value by post-hoc pair-wise comparison using Tukey’s HSD

^c^ *p*-value by two-way repeated ANOVA for the entire QIR and kernel group

Effect of kernel at the same level of iterative reconstruction on FAI: smallest differences in FAI values were found between the quantitative smooth kernel (Qr36) and the vascular smooth kernel (Bv36) with QIR off (delta 1.07 HU). Largest differences in FAI values were found between the sharp vascular kernel (Bv56) and the vascular smooth kernel (Bv36) at QIR4 (delta 19 HU). ANOVA indicated significant main effects of kernels on FAI (*p* < 0.001). In general, the FAI for the vascular kernels decreased with increasing kernel sharpness at each QIR level (see Fig. 2). The quantitative kernel Qr36 showed significant changes in FAI with different QIR levels compared to other kernels (see Table 3 and Fig. 2).

### Image noise and vessel attenuation measurements

Both kernels and iterative reconstruction strength levels were significantly related to noise (*p* < 0.001) (Supplemental Table 1). The noise was significantly reduced at higher QIR levels across all kernels (Supplemental Fig. 1). Higher QIR levels (QIR2 and QIR4) were associated with significantly lower noise, and post-hoc tests confirmed that noise was significantly lower with QIR2 and QIR4 compared to QIR off across all kernels. Among different kernels, The Bv44 and Bv56 kernels were associated with significantly higher noise compared to the Bv36 kernel, while the Qr36 kernel was associated with lower noise (Supplemental Fig. 2). Median attenuation at the origin of the RCA was 865 HU (IQR: 770 HU–904 HU).

### Histogram analysis

**Fig. 3:**
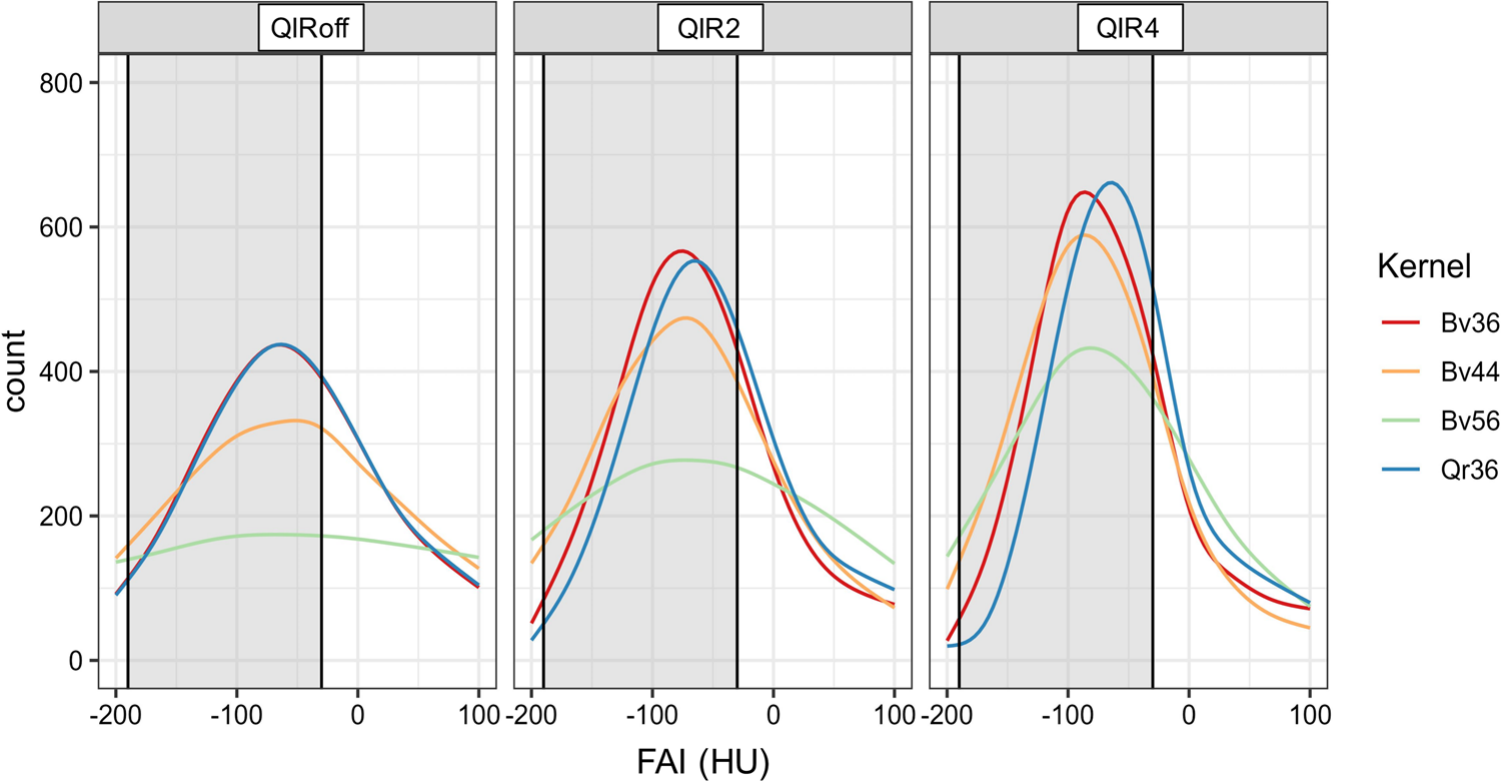
Exemplary histograms of subject no. 10 from the segmented pericoronary adipose tissue volume-of-interest surrounding the proximal right coronary artery (RCA) for the different reconstructions. Gray-shaded areas represent the attenuation interval relevant for FAI from − 190 to −30 HU. Note the different steepness and shift of peaks depending on reconstruction kernel and iterative reconstruction, leading to a different amount of voxels and attenuations included in the FAI analysis

A histogram plot of the distribution of CT values (in HU) within the segmented PCAT VOIs showed bell-shaped curves for each convolution kernel, with a more pronounced narrower peak at increasing QIR levels (QIR off to QIR 4) (Fig. 3).

For the smooth quantitative kernel (Qr36) the position of the peak was independent from the QIR-level. For the smooth, medium and sharp vascular kernels (Bv36, Bv44 and Bv56), the position of the peak shifted towards more negative attenuation values with increasing QIR levels (see Fig. 3).

In the datasets reconstructed without iterative reconstruction (QIR off), the smooth vascular kernel (Bv36) and the corresponding quantitative smooth kernel (Qr36) showed identical histogram curves, which was consistent with the smallest observed FAI differences between these two kernels (see Table 2).

### Edge spread function

**Fig. 4:**
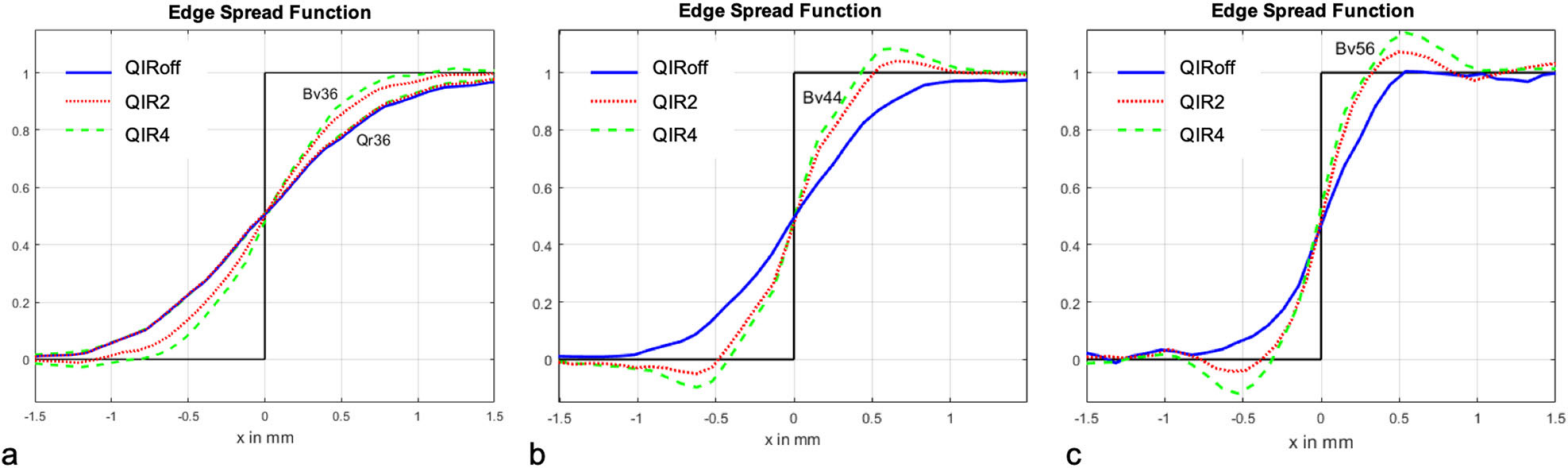
Normalized edge-spread function for the quantitative soft tissue kernel Qr36, the soft tissue kernels Bv36 (**a**), Bv44 (**b**), and the sharp tissue kernels Bv56 (**c**) without iterative reconstruction (QIR off, blue line), and with iterative reconstruction at two different strength levels (QIR 2, dotted red line; QIR 4, dashed green line). The phantom was scanned with identical CT parameters as the subjects. The edge-spread function shows how an edge is represented in the CT image (black function). The figure shows how soft kernels, namely Bv36 and Qr36 (**a**) and QIR off (blue line) smoothen the curve, while sharper kernels, namely Bv44 and Bv56 (**b**, **c**) at QIR4 (dashed green line) lead to a steeper curve

ESFs for the smooth quantitative kernel (Qr36) and the smooth, medium, and sharp vascular kernels (Bv36, Bv44, and Bv56) at different QIR levels (QIR off, QIR2, and QIR4) are shown in Fig. 4.

Without iterative reconstruction (QIR off), the ESF showed an increasingly smoother representation of the edge with decreasing kernel sharpness (blue curves in Fig. 4). The ESFs for Qr36 and Bv36 were identical. The edge-smoothing range on each side of the edge was about 0.6 mm for the sharp kernel, 0.9 mm for the medium kernel, and 1.2 mm for smooth kernels.

With increasing QIR levels (QIR2 and QIR4), the ESF of the quantitative smooth kernel (Qr36) remained unchanged, while the ESFs for the smooth, medium, and sharp vascular kernels became increasingly sharp and showed a pronounced edge overshoot (green and red curves in Fig. 4).

## Discussion

Recent studies suggested that PCAT metabolic activity contributes to the development of coronary atherosclerosis [1, 2] and that FAI measurements reflecting PCAT attenuation in CT may represent a surrogate marker for coronary inflammation [6]. However, CT studies investigating this issue are subject to considerable variation in regard to CT scanner types, scan and contrast media protocols, and image reconstruction parameters [3, 4, 7–10, 13, 20–23, 30, 31].

Our study demonstrated an excellent intra- and inter-reader agreement for FAI measurements in presumably healthy subjects showing no evidence of CAD in CCTA. However, FAI values differed considerably both between subjects (up to 35 HU between individuals) and within subjects depending on both kernels and iterative reconstruction levels (each, *p* < 0.001), with noise changing across different reconstruction levels and indirectly influencing FAI as well. We found differences in FAI attenuation of, on average, 19 HU within the same subjects exclusively related to variable kernels and iterative reconstruction levels.

One of the major findings of this study was the relatively large inter-individual variations in FAI. Inflammation of the pericoronary adipose tissue in at least some of our subjects cannot be excluded, despite the fact that our subjects were otherwise healthy, had a low risk of CAD, no other comorbidities, and showed no CAD in CCTA examinations with excellent image quality. Importantly, these inter-individual variations were related to kernels and iterative reconstruction levels. The sharp vascular kernel (Bv56) without iterative reconstruction (QIR off) showed similar FAI values between subjects (average difference of only 10 HU), suggesting that pericoronary inflammation might not be the reason for these discrepancies in PCAT attenuation, but rather the type of image reconstructions.

Interestingly, the use of different kernels and iterative reconstruction levels led to an increase in this inter-individual difference in PCAT attenuation of up to 35 HU (quantitative smooth kernel Qr36 and QIR4). This difference between subjects substantially exceeds the PCAT attenuation differences of two recent metanalyses in the literature about pericoronary FAI, with reported differences of 6 HU [32] and 5 HU [5] between healthy subjects and patients with CAD. We strongly believe that these reported differences in PCAT attenuation related to reconstruction settings must be considered for follow-up CCTA studies investigating the topic of pericoronary inflammation.

In addition to inter-individual variations, we found considerable intra-individual variations in PCAT attenuation again related to kernels and iterative reconstruction levels. This may be explained, in the absence of evident clinical causes, through the CT value histograms and ESF curves. For the vascular kernels, the FAI decreased with increasing kernel sharpness at the same iterative reconstruction level. This effect is caused by the reduced range of edge smoothing with increasing kernel sharpness. As a result, the CT values measured in the PCAT near the edge of the vessel are less elevated, and the FAI decreases. For the smooth quantitative kernel, FAI increased with increasing iterative reconstruction levels despite of the QIR-independent ESF. This effect is a result of the reduced image noise at increasing QIR strength levels as shown by our results and as previously shown [28]. In the histogram plot, the peak of the CT values in the PCAT is significantly narrower at higher iterative reconstruction levels due to decreasing image noise. By limiting the FAI evaluation to the range of − 190 HU to − 30 HU (which is the standard for FAI assessment [3, 4]), only a part of this peak is asymmetrically averaged, and the FAI thus shifts to higher values as the peak becomes narrower. This effect is less pronounced for the vascular kernels because a complex interplay takes place between reduced image noise (with a tendency towards higher FAI values) and sharper edge display (with a tendency towards lower FAI values) as the iterative reconstruction levels increase.

Both reconstruction kernels and iterative reconstruction algorithms are manufacturer- and often also CT scanner-dependent. Previous PCAT studies included datasets with different reconstruction kernels (for example, a medium soft tissue convolution kernel Bf26 in the study by Dai et al [4], a different medium smooth kernel Bl26 in the study by Xi et al [8], and Bv36 in the study by Moser et al [9]), and the majority did not even report the kernels [3, 4, 7, 10, 20–23, 30, 31]. The same holds true for iterative reconstruction algorithms. Some studies reported the type and strength of iterative reconstruction (for example, Xi et al [8] and Moser et al [9] used sinogram-affirmed iterative reconstruction strength level 3), while others did not report on that issue [3, 4, 7, 10, 20–23, 30, 31]. Reconstruction-specific differences of PCAT may be further aggravated when using monoenergetic image reconstruction of spectral CT data with different keV levels [33]. Mergen et al [33] showed that the FAI of the RCA increased from − 89 ± 8 HU at 55 keV to −77 ± 12 HU at 80 keV. Our findings, despite being limited to a single PCD-CT scanner acquisition and reconstruction parameters, confirm previous evidence by Chen et al [34], who demonstrated with a conventional energy-integrating detector 256-row CT scanner, that PCAT attenuation linearly correlates with iterative reconstructions (*r*-squared > 0.99). These findings further underscore that reconstruction kernels and iterative reconstruction algorithms influence PCAT attenuation, both on new generation and conventional CT scanners, hereby not only expanding the generalizability of our results but also necessitating strong efforts in image acquisition and reconstruction standardization to allow for meaningful interpretations [35].

We used individualized contrast media injection protocols to compensate for inter-individual differences in patient size with the aim of achieving a relatively constant contrast attenuation in the coronary arteries. In our 20 patients, the median attenuation at the level of the origin of the RCA was 865, with an IQR of 770 to 904 at a monoenergetic level of 55 keV. We believe that such relatively constant vessel attenuation is important given the known effect of contrast attenuation on PCAT attenuation, as recently shown in the experimental porcine heart study by Pitteloud et al [36].

The following limitations of our study merit consideration. First, this was a single-center study with a small number of subjects. Second, both the perivascular analysis software and the CT scanner were limited to a single vendor, despite other software tools and scanners being available for these purposes as well. Third, the results were derived from a selected population of presumably healthy subjects, and no data were available for comparison to patients affected by acute and chronic disease. Finally, we cannot exclude pericoronary inflammation in our subjects although we tried to select apparently healthy participants in our study. However, we demonstrated in this study, among other results, large differences also within subjects, only by using different reconstruction settings.

In conclusion, our in-vivo study indicated that PCAT attenuation measured in CT heavily depends on kernels and iterative reconstructions. This further emphasizes the requirement for standardization of both CT data acquisition and reconstruction parameters to meaningfully demonstrate differences both within and across patient populations.

## Supplementary information

ELECTRONIC SUPPLEMENTARY MATERIAL

## Abbreviations

CAD: Coronary artery disease
CCTA: Coronary computed tomography angiography
CI: Confidence interval
ECG: Electrocardiography
ESF: Edge spread function
FAI: Fat attenuation index
HU: Hounsfield unit
ICC: Intraclass correlation coefficient
IQR: Interquartile range
MTF: Modulation transfer function
PCAT: Pericoronary adipose tissue
QIR: Quantum iterative reconstruction
RCA: Right coronary artery
VOI: Volume of interest
