# Supplementary material for: The pericoronary adipose tissue attenuation in CT strongly depends on kernels and iterative reconstructions
Source: Eur Radiol. 2024 Oct 18;35(5):2866–76. doi: 10.1007/s00330-024-11132-5 (PMC12021981; doi:10.1007/s00330-024-11132-5)
Supplement: Supplementary file 1 — ELECTRONIC SUPPLEMENTARY MATERIAL [file 330_2024_11132_MOESM1_ESM.pdf]

The pericoronary adipose tissue attenuation in CT strongly depends on kernels and iterative reconstructions

Electronic Supplementary Material (ESM)

Eur Radiol (2024) Lisi C, Klambauer K, Moser LJ et al.

Supplemental Table 1. Comparison of noise across kernels and iterative reconstruction strength levels.

Kernel	QIR off (HU)	QIR2 (HU)	HSD[1]	QIR4 (HU)	HSD[2]	ANOVA[3]
Bv36	40.6 ± 1.1	37.0 ± 2.3	<0.001	34.0 ± 3.6	<0.001	<0.001
Bv44	43.8 ± 0.8	41.1 ± 1.1	<0.001	38.1 ± 2.2	<0.001	<0.001
Bv56	46.0 ± 0.2	44.9 ± 0.5	<0.001	42.1 ± 0.9	<0.001	<0.001
Qr36	40.6 ± 1.2	35.7 ± 2.6	0.042	31.6 ± 3.2	<0.001	<0.001
ANOVA[3]	<0.001	<0.001	-	<0.001	-	-

QIR Level	Mean FAI of all kernels (HU)	HSD[1]
QIR off	42.7 ± 0.2	-
QIR 2	39.7 ± 0.3	<0.001
QIR 4	36.5 ± 0.5	<0.001

Kernel	Mean FAI of all QIR levels (HU)	HSD[2]
Bv36	37.2 ± 3.7	-
Bv44	41.0 ± 2.8	<0.001
Bv56	44.3 ± 1.8	<0.001
Qr36	36.0 ± 4.4	<0.001

Note. Values are mean and standard deviation unless otherwise specified. ANOVA: analysis of variance, FAI: fat attenuation index, HSD: honestly significant difference test, HU: Hounsfield units, QIR: quantum iterative reconstruction.

[1]QIR off vs. QIR2, QIR off vs. QIR4

[2]Bv36 vs. Bv44, Bv36. vs. Bv56, Bv36 vs. Qr36

[1,2]p-value by post-hoc pair-wise comparison using Tukey's HSD

[3]p-value by two-way repeated ANOVA for entire QIR and kernel group

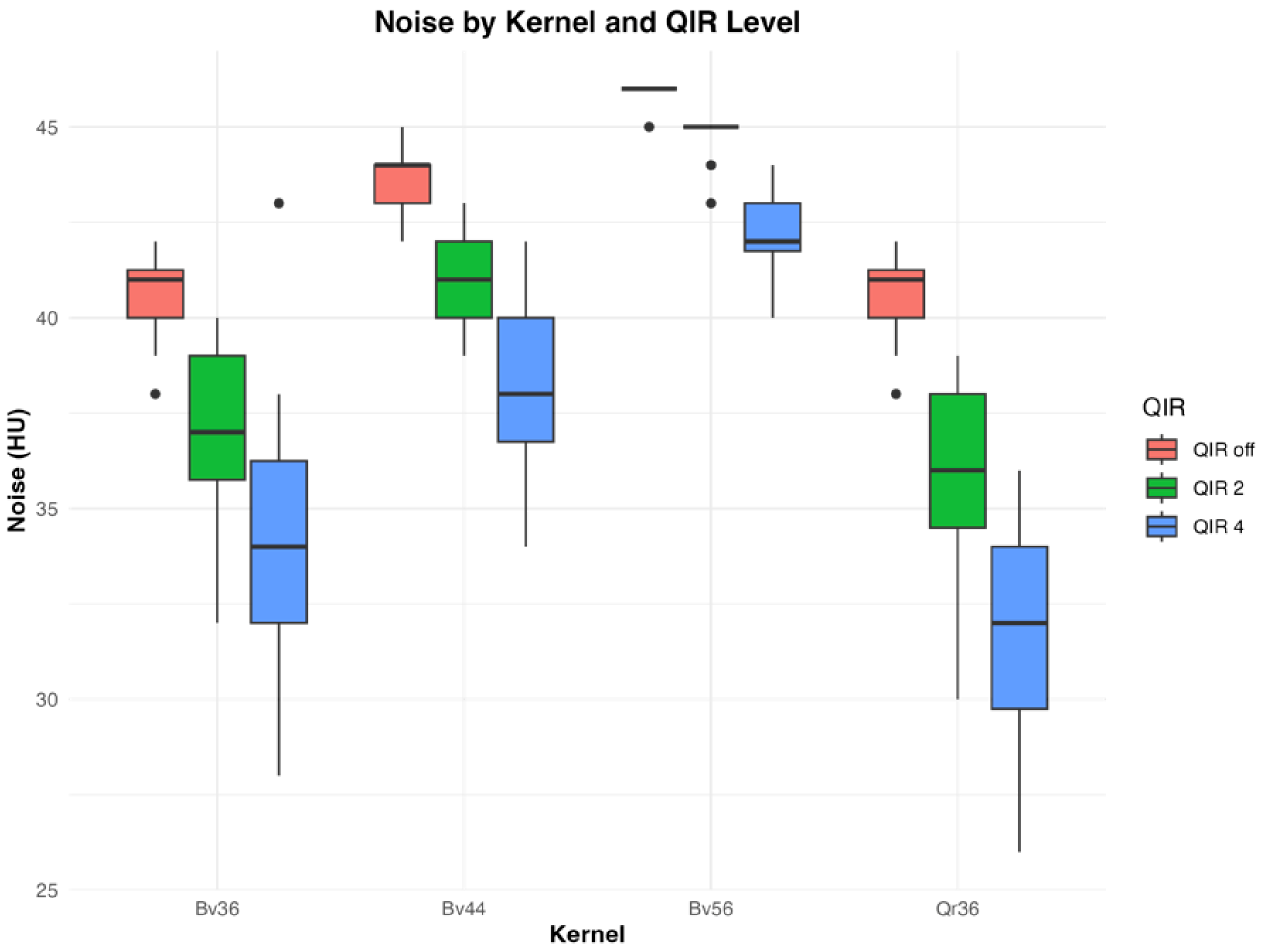

Supplemental Figure 1: Boxplots showing the noise distribution by reconstruction used. As expected, there is considerable effect of iterative strength level and reconstruction kernel on noise

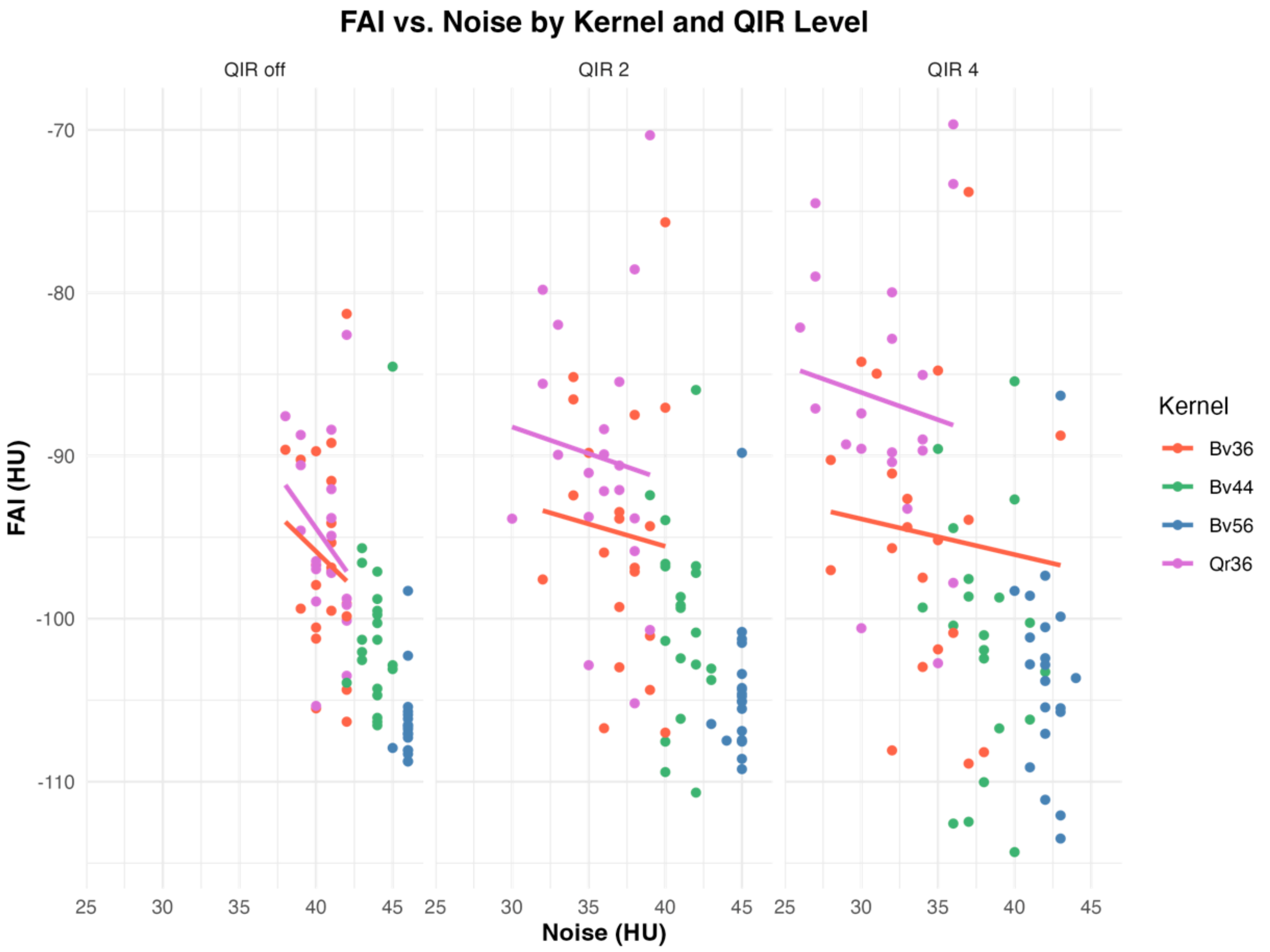

Supplemental Figure 2: Scatterplots showing the individual relationship between kernel, iterative reconstruction and noise on the fat attenuation index (FAI). Note the trend of decreasing FAI values at lower noise levels.
